# Dose, Content, and Context of Usual Care in Stroke Upper Limb Motor Interventions: A Systematic Review

**DOI:** 10.1177/02692155231172295

**Published:** 2023-05-07

**Authors:** Sarah P Newton, Emily J Dalton, Jia Y Ang, Marlena Klaic, Vincent Thijs, Kathryn S Hayward

**Affiliations:** 1 Melbourne School of Health Sciences, University of Melbourne, Melbourne, Australia; 2 Department of Occupational Therapy, Austin Health, Melbourne, Australia; 3 Stroke Theme, Florey Institute of Neurosciences and Mental Health, Melbourne, Australia; 4 Department of Neurology, Austin Health, Melbourne, Australia; 5 Melbourne Medical School, University of Melbourne, Melbourne, Australia

**Keywords:** stroke, upper limb, dose, usual care, rehabilitation

## Abstract

**Objective:**

The objectives of this systematic review were to describe the current dose and content of usual care upper limb motor intervention for inpatients following stroke and examine if context factors alter dose and content.

**Data sources:**

A systematic search (EMBASE, MEDLINE) was completed from January 2015 to February 2023 (PROSPERO CRD42021281986).

**Methods:**

Studies were eligible if they reported non-protocolised usual care upper limb motor intervention dose data for stroke inpatients. Studies were rated using the Johanna Briggs Institute critical appraisal tool. Data were descriptively reported for dose dimensions of time (on task or, in therapy) and intensity (repetitions, repetition/minute), content (intervention type/mode), and context (e.g., severity strata).

**Results:**

Eight studies were included from four countries, largely reflecting inpatient rehabilitation. Time in therapy ranged from 23 to 121 min/day. Time on task ranged from 8 to 44 min/day. Repetitions ranged from 36 to 57/session, and 15 to 282/day. Time on task was lowest in the stratum of people with severe upper limb impairment (8 min/day), the upper limit for this stratum was 41.5 min/day. There was minimal reporting of usual care content across all studies.

**Conclusion:**

Upper limb motor intervention dose appears to be increasing in usual care compared to prior reports (e.g., average 21 min/day and 23 to 32 repetitions/session). Context variability suggests that doses are lowest in the stratum of patients with a severely impaired upper limb. Consistent reporting of the multiple dimensions of dose and content is necessary to better understand usual care offered during inpatient rehabilitation.

## Introduction

Stroke is a leading cause of disability worldwide.^1^ The prevalence of upper limb motor impairment (weakness) within the first week after stroke has been trending downwards from approximately 70% in the early 1990's to 40% to 57% of stroke patients in 2016/2017.^2^ Just under half of those with upper limb impairment early after stroke, remain unable to functionally use their affected upper limb 6 months later,^3^ despite rehabilitation efforts. Together, this highlights why research into upper limb motor interventions early after stroke remains a priority.^4,5^

Usual care reflects the therapy patients receive (dose, content, and context) in routine daily practice^6,7^ which can impact outcomes and is modifiable. Within the evidence base, multiple adjectives are used to describe usual care, such as conventional or routine care.^8^ This likely reflects that usual care is a complex system of interventions that are tailored to an individual.^7^ Understanding usual care is important for researchers and clinicians as it demonstrates what occurs during routine service delivery. The Template for Intervention Description and Replication (TIDieR) checklist was developed to ensure researchers describe all interventions (including usual care) in sufficient detail to allow understanding, monitoring, and replication.^9^ Despite the TIDieR checklist being published in 2014, reporting of usual care data compared to the experimental intervention remains poor.^10^ A lack of reporting, combined with inconsistent terminology for usual care^8^ and the highly variable nature of usual care could be collectively limiting progress in the field of stroke recovery.^7,11^

The dose of usual care (e.g., time in therapy, repetitions)^12^ is considered essential to optimise recovery.^13,14^ Stroke clinical practice guidelines worldwide recommend high doses of usual care therapy to optimise recovery.^15,16^ There is variability in the specificity of dose recommendations for usual care in available guidelines. The United Kingdom guidelines recommend 45 min of each appropriate therapy per day,^16^ while the Australian guidelines recommend three hours of scheduled occupational therapy and physiotherapy daily (time in therapy), with 2 h being active task practice^17^ (time on task). Previous systematic reviews reported that the dose of usual care upper limb motor intervention being provided is significantly less than these guideline recommendations.^18,19^ One review reported the dose to be on average 21 min of time on task in the subacute setting.^18^ It is important to acknowledge that these reviews were completed over 5 years ago and many international guidelines have been updated since their publication. Previous reviews also did not discuss the content or context of usual care. Content refers to what is involved in usual care, such as type of intervention or mode of delivery. Usual care content is tailored to the individual, selected by therapists, and could involve a variety of intervention types and delivery modes.^20^ Context refers to strata characteristics that could impact dose and content, such as upper limb impairment severity. Content and context of usual care are critical factors that enhance our understanding of usual care dose.

This systematic review will provide an update on the current state of usual care upper limb motor intervention dose, content, and context. The aims of this systematic review were to describe the dose and content of usual care upper limb motor intervention for inpatients following stroke and examine if context factors such as upper limb impairment severity alter dose and content observed.

## Methodology

This systematic review was registered on the Prospective Register of Systematic Reviews (CRD42021281986). The Preferred Reporting Items for Systematic Reviews and Meta-Analysis (PRISMA) 2020 statement provided the framework for reporting.^21^

Electronic searches of Ovid EMBASE and Ovid MEDLINE databases were completed from January 1, 2015, to February 23, 2023. The years searched were limited to address the current state of evidence and provide an update from a previous systematic review on the dose of upper limb therapy during inpatient rehabilitation.^18^ The search strategy included Medical Subject Headings and keywords related to stroke, rehabilitation (including occupational therapy and physiotherapy), upper limb, dose, time, accelerometry, and behavioural mapping (see supplemental file STable 1 for full search strategy for each database). A citation-tracking database Web of Science was used, along with hand-scanning the reference lists of included articles to identify additional studies.

All studies identified by the search strategy were uploaded to Covidence and duplicates were removed. Two authors independently screened all titles, abstracts, and full texts for eligibility. Disagreements were resolved by a third author not involved in the initial screening.

Studies were screened against predetermined eligibility criteria. Studies that included adult (≥18 years) stroke survivor participants who received acute or subacute inpatient care and reported usual upper limb motor care (e.g., standard care, routine care, conventional care) were eligible. Eligible studies had an English abstract and were required to report usual care upper limb motor intervention dose in minutes, hours, repetitions, or activity counts. Motor interventions not included in the Pollock et al., (2014) systematic review were excluded given the potential variability of usual care content. Study designs included were observational and interventional studies such as pre–post interventions, or randomised control trials that reported usual care dose data for the control group (if control received usual care only). Non-stroke cohorts were excluded. If a cohort included stroke and stroke-like conditions, the corresponding author was contacted for individual data of the stroke participants only. Studies that included participants from community settings were excluded as models of community rehabilitation differ in dose and scheduling as compared to inpatient rehabilitation. Interventional studies that protocolised usual care i.e., predefined dose and content, were excluded.

Eligible studies were appraised for quality using The Joanna Briggs Institute critical appraisal tool for analytical cross-sectional studies.^22^ Given the variability in study design, three authors met to define the justification for rating studies using the checklist for the current research question to aid transparency in the application (see supplemental file STable 2). Appraisals were completed by the primary author, and a second author independently rated over two-thirds of the studies. The two authors met to compare ratings and a consensus was reached through discussion.

A custom-designed excel spreadsheet was developed for data extraction. Data were extracted by the primary author and all data were independently cross-checked by a second author. Data extracted included study characteristics, participant characteristics, dose, and content of therapy as outlined in Table 1. Only the control intervention group data at baseline were extracted for interventional studies regardless of whether the experimental group also received usual care. The corresponding author was contacted if additional data were required or if data needed to be clarified.

**Table 1.** Data extraction summary.

**Data category**	Sub-category data	Classification of data
Study characteristics	Title Author Country research conducted	NA
	Setting Year data collected	
	Sample size Recording method	
Participant characteristics	Age Sex Stroke type	
	Time post stroke	Hyperacute: < 24 h Acute: 24 h–<7 days Early subacute: 7 days–< 3 months Late subacute: 3 months–< 6 months Chronic: > 6 months
	Stroke severity: NIHSS	Minor: 1–4 Moderate: 5–15 Moderate/severe: 16–20 Severe: 21–42
	Upper limb deficits	
	FMA-UL	Mild: 42–66 Moderate: 29–41 Severe: 0–28
	MAS-UL	Mild: 14–18 Moderate: 3–13 Severe: 1–2
Dose	Multidimensional dose articulation framework	Days Sessions Time in therapy Time on task Intensity
Content	Adjective to describe intervention Intervention described as guideline care	
	Intervention described using the ICF	Body functions and structures Activities Participation
	Intervention described using type	
	Intervention described using mode of delivery	Supervised Unsupervised 1:1 Group

*Note*. FMA-UL: Fugl–Meyer Assessment Upper Limb; MAS-UL: Motor Assessment Scale Upper Limb; NIHSS: National Institutes of Health Stroke Severity Scale; NA: Not Applicable; ICF: International Classification of Functioning, Disability and Health.

Demographic characteristics (e.g., age, sex, stroke type, time post-stroke, stroke severity, and upper limb deficits) were extracted and classified according to current field standards (Table 1). Time post-stroke was classified using international consensus definitions developed from the first Stroke Recovery and Rehabilitation Roundtable (SRRR).^23^ Upper limb severity was determined using the Fugl–Meyer Assessment Upper Limb (FMA-UL) defined levels^24^ and the Motor Assessment Scale Upper Limb (MAS-UL) scores previously reported.^25^ Where both scales were used, the more common scale (FMA-UL) was chosen to represent the upper limb severity level. Stroke severity was reported if the National Institutes of Health Stroke Scale (NIHSS) was included and participants were defined as mild, moderate, moderate/severe, or severe.^26^ If a study reported a different classification system, the relevant classification was used. Data were classified as not reported when no measure was used to report upper limb or stroke severity.

All available dimensions of dose data from the multidimensional dose articulation framework^12^ were extracted. The primary dimensions of interest were: ‘time in therapy’ (minutes), ‘time on task’ (minutes), and intensity (number of repetitions and repetitions per minute) of upper limb training across a single session or day of training. Where applicable, data presented in hours were converted to minutes. If available, upper limb training provided by discipline (occupational therapy or physiotherapy) was extracted. Data related to time spent in occupational therapy and physiotherapy were combined to report total time across a day. All results are reported descriptively and the ranges of each dose dimension are stated.

Content of usual care interventions was extracted across five categories that were determined through content analysis: (1) adjective to describe the intervention e.g., usual care; (2) whether the intervention was described as guideline care (yes/no); (3) if the intervention was described using the International Classification of Functioning, Disability, and Health (ICF) (yes/no)^27^; (4) if the intervention was described (yes/no) and by type (yes/no), e.g., task-specific; (5) if the intervention mode of delivery (e.g., supervised) was described (yes/no). See supplemental file STable 3 for details on how these categories were developed and data extracted.

Context data within a study that were stratified by upper limb severity or, cognitive or language ability were extracted and analysed separately. If context data could be used to inform overall dose data, context strata (upper limb severity or cognitive/language ability) were averaged to calculate the total dose. In studies where the overall upper limb impairment level was severe, these data were grouped with the severe upper limb impairment stratum. The corresponding author was contacted to request additional context data if required.

## Results

**Figure 1.**
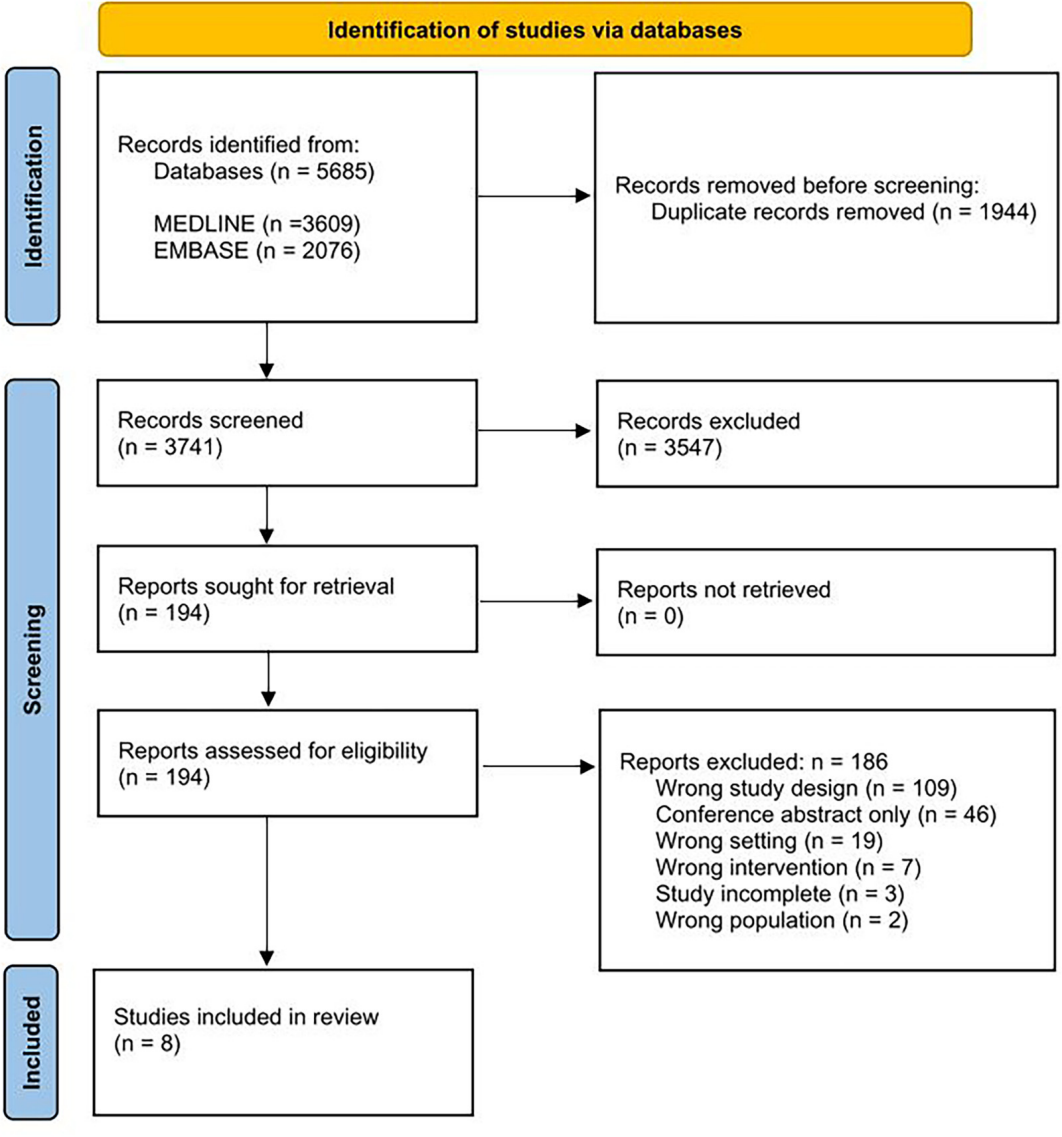
PRISMA 2020 flow diagram.

The search yielded 5685 studies (EMBASE = 2076, MEDLINE = 3609). The total yield was 3741 studies once duplicates had been removed. A total of 3547 titles and abstracts were excluded and 194 full-text articles were screened for eligibility. At full-text screening, 186 were excluded (wrong study design n=109, conference abstract only n=46, wrong setting n=19, wrong intervention n=7, study incomplete n=3, wrong population n=2). The most common query in full-text was reporting of usual care dose. More than eight studies required detailed discussion which led to their exclusion. The final yield of included studies was eight. No additional studies were identified through hand-searching. Three corresponding authors were contacted for additional information and two responded: one related to study population^28^ and one related to usual care dose.^29^ See Figure 1 for PRISMA flow diagram.

Study characteristics and participant characteristics are reported in Table 2. Study locations spanned Australia (n=4),^28,30–32^ Singapore (n=2),^29,33^ Netherlands (n=1),^34^ and the United Kingdom (n=1).^35^ Most studies were completed on the subacute ward (n=6),^28,29,31–34^ with one study completed on the acute ward^35^ and one completed on a mixed acute and subacute ward.^30^ The average time since stroke onset ranged from 3.1^30^ to 43.5 days.^34^ One study^30^ was considered in the acute phase^23^ of recovery at time of recruitment, while the remaining seven were in the early subacute phase.^23^ Half of the studies aimed to investigate upper limb usual care,^29,30,33,34^ while the remaining were interventional studies that incidentally reported upper limb usual care dose.^28,31,32,35^ Studies collected dose data via therapist recording (n=4),^31,33–35^ behavioural mapping (n=3),^28,29,32^ or a mixed method of therapist, patient or family counting (n=1).^30^ One study collected behavioural mapping and accelerometry data.^29^ Only two studies stated an explicit aim to report on the content of usual care.^30,34^

**Table 2.** Study characteristics and participant demographics.

**Author, Year Published, Country**	*Trial design*	*Recording method*	*Setting^*	*Therapy days/week*	*Therapy sessions*	*Year data collected*	*Sample*	*Male: female*	*Age*	*Ischemic: haemorrhagic stroke*	*Days post-stroke*	*Stroke severity*	*Upper limb severity*
Broderick 2021, United Kingdom	Pre-post clinical trial	Therapist recorded	Acute			2019	20	6:14	63^b^	14:6	8^b^	Moderate	Moderate
Chin 2019, Singapore	Cross-sectional cohort	Behaviour mapping and accelerometry	Subacute rehabilitation			NR	12	7:5	61^bd^	7:5	21^bd^	Moderate^d^	Mild^d^
Chin 2020, Singapore	Cross-sectional cohort	Therapist recorded	Subacute rehabilitation			2016–2017	60	45:15	61^ac^	45:15^c^	18.7^ac^	Moderate^c^	Moderate^c^
de Jong 2018, Netherlands	Retrospective analysis from randomised control trial	Therapist recorded	Rehabilitation	5	2-3^c^ /day	2008–2010	46	27:19	57.5^a^	37:9	43.5^a^	NR	Severe
Flynn 2022, Australia	Pre-post clinical trial	Behaviour mapping	Rehabilitation			2016	7	3:4	68^a^	5:2	32^a^	NR	NR
Horsley 2019, Australia	Randomised control trial	Therapist recorded	Rehabilitation	5	23 total	2015–2017	25	12:13	68.5^a^	23:2	24.9^a^	NR	Severe
Schneider 2019, Australia	Pre-post clinical trial	Behaviour mapping	Rehabilitation			NR	8	NR	NR	NR	NR	NR	NR
Vratsistas-Curto 2021, Australia	Longitudinal cohort	Mixed, self, therapist, or family	Mixed acute and subacute ward		1.5^b^ /week	2011–2013	99	52:47	75.8^a^	82:17	3.1^a^	NR	NR

*Note.* NR: Not Reported. Setting as described by the paper^, note terminology used may be different depending on the country. Mean^a^. Median^b^. Data calculated for review^c^_._ Additional information gathered from the corresponding author^d^

The quality rating for the majority of studies was high; with 88% scoring greater than or equal to seven out of eight (individual results reported in supplemental file STable 4). One study^28^ scored a low-quality rating of three out of eight as the participants of this study were staff not stroke survivors. Therefore, some questions, such as *were standard criteria used to measure the condition* (condition being stroke), could not be applied effectively.

### Dose

Seven studies reported dose in time (minutes).^28,29,31–35^ Five studies reported dose as time in therapy^28,29,32,33,35^ and four reported time on task.^28,29,31,34^ Four studies also reported dose as repetitions.^28,30,32,35^ Individual study dose data are presented in Table 3.

**Table 3.** Reported dose of usual care according to the multidimensional dose articulation framework.

**Study**		Time, min	Repetitions/day	Repetitions/session	Repetitions/minutes
Broderick	*All*	*In therapy 23.3^b^/day*	*15^b^*	*36^b^*	*0.64^b,c^*
	OT	-	-	-	-
	PT	-	-	-	-
Chin 2019^d^	*All*	On task: 44.4^b,c^/day *In therapy: 120^b,c^/day*	*-*	*-*	*-*
	OT	On task: 22.2^b,c^/day In therapy: 55^b^/day	-	-	-
	PT	On task: 22.2^b,c^/day In therapy: 65^b^/day	-	-	-
Chin 2020	*All*	*In therapy 120.8^a,c^/day*	*-*	*-*	*-*
	OT	In therapy: 57^a,c^/day	-	-	-
	PT	In therapy: 63.8^a,c^/day	-	-	-
de Jong	*All*	*On task^,^ 8^b^/day*	*-*	*-*	*-*
	OT	On task: 6^b^/day	-	-	-
	PT	On task: 2^b^ /day	-	-	-
Flynn	*All*	* In therapy 55^a^/day*	*282^a^*	*-*	*5^a^*
	OT	-	-	-	-
	PT	-	-	-	-
Horsley	*All*	*On task: 41.52^b,c^/day*	*-*	*-*	*-*
	OT	-	-	-	-
	PT	-	-	-	-
Schneider	*All*	*-*	*-*	*^−^*	*^−^*
	OT	In therapy 52.33^a,d^/session On task19.13^a,d^ /session	-	60^a,d^	2.4^a,d^
	PT	-	-	-	-
Vratsistas-Curto	*All*	*-*	*68.5^b^*	*56.9^b^*	*-*
	OT	-	-	-	-
	PT	**-**	**-**	**-**	**-**

*Note.* OT: Occupational therapy, PT: Physiotherapy, Mean^a^, Median^b^, Data calculated by authors of this review^c^, Additional information gathered from author^d^.

Of the studies reporting time in therapy, the dose ranged from a median of 23.3 min per day^35^ to a mean of 120.8 min per day.^33^ Two studies^29,33^ reported time in therapy during occupational therapist versus physiotherapist-led sessions. One study reported a median of 55 and 65 min per day of occupational therapy and physiotherapy, respectively,^29^ and another reported a mean of 57.0 and 63.8 min per day of occupational therapy and physiotherapy, respectively.^33^

Regarding time on task, one study reported a median of 6 min on task in occupational therapy and a median of 2 min on task in physiotherapy per day,^34^ while another reported a median of 22.2 min per day on task in each of occupational therapy and physiotherapy-led sessions.^29^ Combined time across disciplines ranged from a median of 8 min^34^ to a median of 44.4 min^29^ per day on task.

For the studies that reported the intensity of dose in repetitions, some reported repetitions per session, while others reported repetitions per day. Repetitions per session ranged from a median of 36 repetitions^35^ to a median of 56.9 repetitions,^30^ while repetitions per day ranged from a median of 15 repetitions^35^ to a mean of 282 repetitions.^32^ Three studies reported intensity as the rate of repetitions per unit of time.^28,32,35^ One reported a median 0.64 repetitions per minute,^35^ one reported a mean of 2.4 repetitions per practice minute,^28^ and another reported a mean of 5 repetitions per minute.^32^

### Content

The adjective used to describe usual care intervention varied across studies. Only two studies referenced stroke guidelines in their usual care intervention description.^28,34^ Five studies described their intervention using ICF categories^28,30–32,34^: four referred to body functions and structures, and activities^28,30–32^; while one study also referred to participation.^34^ Only one study reported proportion of time spent by discipline in each ICF category^34^: occupational therapists spent 1% of their treatment time at the participation level, 41% at the activities level, and 47% at the level of body functions and structures; while physiotherapists spent 2% of their treatment time at the participation level, 14% at the activities level, and 79% at the level of body functions and structures. Five studies^28,30,31,34,35^ described content by type of intervention (ranging from one to three interventions), while four studies reported content by active or passive tasks.^28,31,32,34^ Two studies did not describe the content of usual care.^29,33^ Of the six studies that reported mode of delivery,^28,30–32,34,35^ only two reported the use of unsupervised or self-directed practice.^30,35^ Table 4 outlines the reported content of usual care.

**Table 4.** Reported content of usual care.

**Reference**	Broderick 2021	Chin 2019	Chin 2020	de Jong 2018	Flynn 2022	Horsley 2019	Schneider 2019	Vratsistas-Curto 2021
**Adjective to describe intervention**	“standard care” “conventional therapy”	“people in rehabilitation”	-	“conventional therapy”	“routine clinical practice”	“usual upper limb care”	“usual care upper limb rehabilitation”	“usual rehabilitation”
**Intervention described as guideline care**	-	-	-	Y	-	-	Y	-
**Intervention described using ICF model**	-	-	-	Y	Y	Y	Y	Y
Body functions and structures	-	-	-	Y	Y	Y	Y	Y
Activities	-	-	-	Y	Y	Y	Y	Y
Participation	-	-	-	Y	-	-	-	-
**Intervention described using type**	Y	-	-	Y	-	Y	Y	Y
Task-specific	-	-	-	-	-	Y	-	Y
Strength training	-	-	-	Y	-	Y	-	-
Electrical stimulation	-	-	-	-	-	Y	Y	-
Mental practice	-	-	-	Y	-	-	Y	-
GRASP	Y	-	-	-	-	-	-	-
**Intervention described using mode of delivery**	Y	-	-	Y	Y	Y	Y	Y
Supervised	Y	-	-	Y	Y	-	Y	Y
Unsupervised/self-directed	Y	-	-	-	-	-	-	Y
1:1	-	-	-	-	-	Y	-	Y
Group	-	-	-	-	-	Y	Y	Y

*Note.* Y given to study when described usual care content in aims, methods or results, - not reported. This excluded content description of intervention groups, GRASP: Graded Repetitive Arm Supplementary Program.

### Context

Three studies stratified dose data by upper limb severity,^29,32,33^ and one study stratified dose data by cognitive or language ability.^30^ An additional two studies included only participants with a severely impaired upper limb as per their inclusion criteria.^31,34^ No studies reported different content e.g., type of intervention, by severity of upper limb impairment, or any other stratification variable.

The stratum receiving the lowest dose was those with severe upper limb impairment. Time on task ranged from a median of 8 min^34^ to a median of 41.5 min per day.^31^ Only one study reported time on task and time in therapy for different upper limb strata in the same setting.^29^ The severe stratum received a median of 33.0 min of time on task (median of 130 min of time in therapy) per day of occupational therapy and physiotherapy combined.^29^ In contrast, the mild stratum received a median of 67.2 min of time on task (median of 50 min of time in therapy) per day of occupational therapy and physiotherapy combined.^29^ Note the lower time in therapy compared to time on task was due to some mildly impaired patients not receiving occupational therapy for their upper limb.

One study reported time in therapy and intensity for the total sample compared to a severe upper limb stratum.^32^ The severe stratum received a mean of 59 min time in therapy per day, with a mean of 265 repetitions performed. This resulted in an intensity of 4 repetitions per minute. In comparison, the total sample received a mean of 55 min time in therapy per day, with a mean of 282 repetitions performed. This resulted in an intensity of 5 repetitions per minute.^32^

One study provided stratified dose data by cognitive or language impairment status.^30^ The total sample (inclusive of patient with cognitive or language impairment) received a median of 56.8 repetitions per session and a median of 68.5 repetitions per therapy day. Comparatively, the stratum without cognitive or language impairment received a median of 69.9 repetitions per session and a median of 77.9 repetitions per therapy day.^30^

## Discussion

This systematic review demonstrated that research into usual care dose and content for upper limb motor interventions after stroke remains scarce. Only eight studies were identified (between January 2015 and February 2023), with most collecting data within a subacute inpatient environment. The maximum time in therapy per day (120.8 min)^33^ reported was five times greater than the minimum time in therapy (23.3 min).^35^ The maximum time on task per day (44.4 min)^29^ was also five times the minimum time on task (8 min).^34^ Repetitions ranged on average from 36^35^ to 57^30^ per session, while per day ranged from 15^35^ to 282.^32^ Severe upper limb impairment and reduced cognitive or language ability were found to impact the dose of upper limb usual care. The findings of this review demonstrate that there is considerable variability in dose on average, which means a given individual could be receiving more, but also far less than described. This finding has important implications for clinical trial design, especially multisite trials, that adopt a usual care control group.

Time on task and number of repetitions have increased since previous systematic reviews on this topic.^18,19^ Hayward and Brauer (2015) reported on average 21 min of time on task per day during subacute inpatient rehabilitation across included studies. They also reported a narrow range of 23 to 32 repetitions per session. Serrada et al., (2016) reported 7.9 min of time on task per session, however, this review only included studies that collected data in an acute inpatient setting. While it is encouraging to see that the dose of upper limb motor intervention provided in usual care is increasing, there remains a considerable gap between the dose of usual care reportedly provided and the recommended two hours of active practice across occupational therapy and physiotherapy as per the ‘living’ Australian clinical practice guidelines.^17^ Narrowing the evidence-practice gap may help maximise upper limb recovery for people after stroke.

The variability in dose identified in this review was complicated by inconsistencies in how dose data were reported e.g., either time on task or time in therapy as well as using mean or median summary statistics. We attempted to apply the multidimensional dose articulation framework^12^ to examine dose in this review. While we recognise this framework was published after most studies included in this review were conducted, few studies reported more than one dimension of dose e.g., time in therapy, time on task, and intensity. Furthermore, studies poorly reported how they calculated dose, as well as fidelity of recording and training of data collectors recording usual care dose. Future research into usual care should consider dose as a multidimensional construct with data collected for all dose dimensions to enable data pooling and comparison between studies and sites. Adopting a consistent approach to data collection and reporting is a necessary step to advance the science of usual care in clinical trials and our understanding of how dose may mediate stroke recovery.

There was minimal reporting of the content of upper limb motor intervention across studies. Few studies acknowledged the complex system of usual care and its multiple dimensions. This underreporting of usual care is also true of interventional studies across the field of stroke rehabilitation.^8^ Previous reviews have demonstrated that the control group is insufficiently described compared to the experimental group^10,36^ despite TIDieR guidelines.^9^ Thorough descriptions of content are important to enhance our understanding of dose. One study included in this review provided a description of how one repetition varied between an impairment and activity-based task, which highlights how content can impact the total number of repetitions documented.^32^ As repetitions are not all created equal, it brings into question if repetitions are the most reliable dimension of dose. Additionally, there was variation in how content was recorded. Some studies used the ICF, while others described if the intervention was active or passive. Given the variability within the limited information available, it was challenging to draw meaningful conclusions about content.

Only three studies used the adjective ‘usual care.’ This finding is consistent with other stroke rehabilitation literature that observed the adjective used to report content is not only highly variable,^8^ but used synonymously to describe both protocolised and non-protocolised content, despite having vast differences in practice. Consistent use of an adjective to describe content is important given the prevalence of non-protocolised usual care as the control intervention in many clinical trials.^36^ An alternative adjective is also required for protocolised usual care.^8^ Reducing variability can aid in comparison of an intervention to a clinically realistic usual care control group.

While variability in content for different strata was not described, the large range of doses articulated in the literature could be attributed to contextual factors of patient characteristics, particularly the severity of upper limb impairment. In a sample of people with severe upper limb impairment, de Jong and colleagues^34^ reported the lowest time on task (8 min), while Horsley and colleagues^31^ had a comparatively higher time on task (41.5 min per day). To help understand the impact of severity, we can draw on one study that recruited participants into three upper limb severity strata.^29^ Time on task was only 33.0 min for the severe stratum, while the mild stratum received almost double the dose at 67.2 min per day.^29^ This suggests that while severity may impact dose, engaging people in therapy after stroke with severe upper limb impairment was possible. There was also a considerable difference between time in therapy versus time on task. This difference was particularly notable in the severe stratum in Chin et al., (2019). This could be due to clinicians providing different content of intervention to patients with severely impaired upper limbs.^37^ The interventions provided to patients with severely impaired upper limb have been previously reported to be passive, such as oedema and musculoskeletal management i.e., positioning, ranging, and non-functional electrical stimulation.^37^ Passive interventions were often excluded in documenting of usual care dose in studies included in this review. Future studies should report the content of therapy in a more nuanced way (including passive and active time in therapy) as this will allow researchers and clinicians to better understand the usual care intervention content provided to patients with different upper limb impairment severities. Only one study reported the impact of cognition and language deficits resulting in a lower dose of upper limb therapy.^30^ Interestingly, no other studies stratified by any other context factors such as sensory impairment.

There are several limitations to the current review. Firstly, this review only included studies that reported usual care dose and then considered their description of intervention content. Given studies that reported on the content of usual care without dose information were excluded, the described usual care content may be underrepresented. While our review included studies published between 2015 and 2023, data collection dates ranged from 2008 to 2019, which could indicate that some data were more than a decade old. Regular investigation of usual care remains important to understand current practice for clinical trial design, which is likely to evolve with time, new evidence, and clinical care practices. We acknowledge that different centres have different models of usual care with differing factors impacting who can gain access to rehabilitation.^38^

In conclusion, usual care rehabilitation is a complex multidimensional system. There continues to be little information provided about the dose and content of usual care to inform clinical and research protocols of upper limb motor interventions post-stroke. In future studies, researchers should use the term ‘usual care’ when describing non-protocolised intervention and report on the multiple dimensions of dose consistent with multidimensional dose articulation framework to allow for comparison between studies. While it is promising to see an increase in usual care dose reported over the past eight years, further increases are needed to optimise upper limb recovery of stroke patients.

**Clinical message**

- Dose of upper limb usual care provided to stroke patients is highly variable.
- Time on task ranged from 8 to 44 min/day, while repetitions ranged from 15 to 282/day.
- While the content of usual care is largely unknown, context factors, such as severity and cognition may impact dose received.

## Supplemental Material

sj-docx-1-cre-10.1177_02692155231172295 - Supplemental material for Dose, Content, and Context of Usual Care in Stroke Upper Limb Motor Interventions: A Systematic Review Click here for additional data file. Supplemental material, sj-docx-1-cre-10.1177_02692155231172295 for Dose, Content, and Context of Usual Care in Stroke Upper Limb Motor Interventions: A Systematic Review by Sarah P Newton, Emily J Dalton, Jia Y Ang, Marlena Klaic, Vincent Thijs and Kathryn S Hayward in Clinical Rehabilitation
