# Supplementary material for: Dose, Content, and Context of Usual Care in Stroke Upper Limb Motor Interventions: A Systematic Review
Source: Clin Rehabil. 2023 May 7;37(11):1437–50. doi: 10.1177/02692155231172295 (PMC10492439; doi:10.1177/02692155231172295)
Supplement: sj-docx-1-cre-10.1177_02692155231172295 - Supplemental material for Dose, Content, and Context of Usual Care in Stroke Upper Limb Motor Interventions: A Systematic Review [file sj-docx-1-cre-10.1177_02692155231172295.docx]

**Supplemental file:**

**Title:** Dose, content, and context of usual care upper limb motor interventions for stroke inpatients: A systematic review

**Corresponding author**

Kathryn Hayward

Harold Stokes Building, Austin Hospital, Heidelberg, Australia

Email: kate.hayward@unimelb.edu.au

Telephone: +61 03 9035 7293

Twitter: @kate_hayward_

**STable 1**: Search strategy Ovid EMBASE and Ovid MEDLINE

|  | Population | Intervention | Intervention | Outcome |
| --- | --- | --- | --- | --- |
| Ovid  EMBASE | **1.** exp Cerebrovascular Disease/  **2.** stroke$.mp.  **3.** cva$.mp.  **4.** cerebrovascular$.mp.  **5.** cerebral vascular$.mp.  **6.** (poststroke or post-stroke or cerebrovasc$ or brain vasc$ or cerebral vasc$ or  apoplex$ or SAH).mp.  **7.** ((brain$ or cerebr$ or cerebell$ or intracran$ or intracerebral or vertebrobasilar) adj5  (isch?emi$ or infarct$ or thrombo$ or emboli$ or occlus$)).mp.  **8.** ((brain$ or cerebr$ or cerebell$ or intracerebral or intracranial or subarachnoid) adj5  (haemorrhage$ or hemorrhage$ or haematoma$ or hematoma$ or bleed$)).mp.  **9.** exp hemiplegia/ or exp paresis/  **10.** (hemipleg$ or hemipar$ or paresis or paretic).mp.    **11**. 1 or 2 or 3 or 4 or 5 or 6 or 7 or 8 or 9 or 10 | **12**. exp Physiotherapy/  **13.** physical therap$.tw  **14.** Physiotherap$.mp.  **15.** Exp Occupational therapy/  **16.** Occupational Therap$.mp.  **17.** exp physical activity/  **18.** activit$.mp.  **19.** exp Rehabilitation/  **20.** rehabilitat$.mp.  **21.** (motor relearn$ or motor learn$).mp.  **22.** bobath.mp.  **23**. exp Motor activity/  **24.** exp training/  **25.** exp exercise /    **26.** 12 or 13 or 14 or 15 or 16 or 17 or 18 or 19 or 20 or 21 or 22 or 23 or 24 or 25 | **27.** exp upper limb/  **28.** (upper limb$ or upper extremit$ or arm or shoulder or hand or axilla or elbow$ or forearm$ or finger$ or wrist$).mp.    **29.** 27 or 28 | **30.** behavio* map*.mp.  **31.** Treatment time.mp.  **32.** exp time/  **33.** therap* time .mp.  **34.** time taken .mp.  **35.** time spent.mp.  **36.** Session time.mp.  **37.** Activ$ time.mp.  **38.** exp exercise intensity/  **39.** intensity.mp.  **40.** repetit*.mp.  **41.** exp dose/  **42.** Dose.mp.  **43.** exp accelerometry/  **44.** exp accelerometer/  **45.** exp activity tracker/  **46.** exp behavioral observation/  **47.** exp observation/  **48.** Observation$ technique$.mp.  **49.** Exp treatment duration/    **50.** 30 or 31 or 32 or 33 or 34 or 35 or 36 or 37 or 38 or 39 or 40 or 41 or 42 or 43 or 44 or 45 or 46 or 47 or 48 or 49 |
| Ovid  MEDLINE | 1. exp Cerebrovascular Disorders/  2. stroke$.mp.  3. cva$.mp.  4. cerebrovascular$.mp.  5. cerebral vascular$.mp.  6. (poststroke or post-stroke or cerebrovasc$ or brain vasc$ or cerebral vasc$ or  apoplex$ or SAH).mp.  7. ((brain$ or cerebr$ or cerebell$ or intracran$ or intracerebral or vertebrobasilar) adj5  (isch?emi$ or infarct$ or thrombo$ or emboli$ or occlus$)).mp.  8. ((brain$ or cerebr$ or cerebell$ or intracerebral or intracranial or subarachnoid) adj5  (haemorrhage$ or hemorrhage$ or haematoma$ or hematoma$ or bleed$)).mp.  9. exp Hemiplegia/ or exp Paresis/  10. (hemipleg$ or hemipar$ or paresis or paretic).mp.        11. 1 or 2 or 3 or 4 or 5 or 6 or 7 or 8 or 9 or 10 | 12. exp Physical Therapy Modalities/   13. physical therap$.mp.  14. Physiotherap$.mp.  15. Exp occupational therapy/  16. Occupational Therap$.mp.  17. Physical activity.mp.  18. Activit$.mp.  19. exp Rehabilitation/  20. rehabilitat$.mp.  21. (motor relearn$ or motor learn$).mp.  22. Bobath.mp.  23. exp motor activity/  24. training.mp.  25. exp exercise/    26. 12 or 13 or 14 or 15 or 16 or 17 or 18 or 19 or 20 or 21 or 22 or 23 or 24 or 25 | 27. exp Upper Extremity/    28. (upper limb$ or upper extremit$ or arm or shoulder or hand or axilla or elbow$ or forearm$ or finger$ or wrist$).mp.    29. 27 or 28 | 30. behavio* map*.mp.  31. Treatment time.mp.  32. exp time/  33. therap* time.mp.  34. time taken.mp.  35. time spent.mp.  36. Time use {Including Related Terms}  37. Session time.mp.  38. Activ$ time.mp.  39. Exercise intensity.mp.  40. intensity.mp.  41. repetit*.mp.  42. Dose.mp.  43. exp accelerometry/  44. Accelerometer.mp.  45. activity tracker.mp.  46. Behavio* observation.mp.  47. exp observation/  48. Exp behavior observation techniques/  49. Observation$ technique$.mp.  50. exp duration of therapy/  51. Treatment duration.mp.  **52.** 30 or 31 or 32 or 33 or 34 or 35 or 36 or 37 or 38 or 39 or 40 or 41 or 42 or 43 or 44 or 45 or 46 or 47 or 48 or 49 or 50 or 51 |

**STable 2**: Quality appraisal – Justification criteria

| JBI question | Interpretation used for systematic review |
| --- | --- |
| Were the criteria for inclusion in the sample clearly defined? | Full description of inclusion and exclusion provided in main text or supplementary.  Stroke participants with upper limb impairment were of interest. |
| Were the study subjects and the setting described in detail? | Report on **both** subjects and setting to score a yes.  Subjects: Description of patient characteristics including but not limited to age, sex, type of stroke, time since onset  Setting: Must report inpatient or outpatient setting may include acute or subacute setting and if the study was conducted across multiple sites. |
| Was the exposure measured in a valid and reliable way? | The exposure was dose of usual care upper limb intervention.  Demonstrated consistency across observers e.g., staff training, or definition of repetition. |
| Were objective, standard criteria used for measurement of the condition? | The condition is stroke.  Reported as “confirmed,” “diagnosed,” physician determined reported as infarct or heamorrhage, or confirmed on CT or MRI to score a yes. |
| Were confounding factors identified? | Description of patient characteristics provided e.g., age, time post-stroke, upper limb severity, cognitive ability. |
| Were strategies to deal with confounding factors stated? | Scored yes if purposefully recruiting e.g., narrowed inclusion criteria, stratified groups, or had a reasonably broad sample such that it was reasonable not to have stated confounding factors. |
| Were the outcomes measured in a valid and reliable way? | The outcome was the assessment of upper limb motor deficits. |
| Was appropriate statistical analysis used? | In relation to only the usual care dose data i.e., not content data or between-group comparison. Data were represented as mean or median. |

**STable 3**: Content extraction

| **Reference** | **Adjective to describe intervention** | **Intervention described as guideline care** | **Intervention described using ICF model** | **Intervention described using type** | **Intervention described using mode of delivery** |
| --- | --- | --- | --- | --- | --- |
| Broderick 2021 | "Conventional therapy was not intended to be altered by, nor incorporate device use. To confirm this, we also conducted a prospective audit of therapy content and duration in a separate cohort of patients with similar characteristics, receiving standard care only" | - | - | "We also recorded whether, as part of conventional therapy, participants had an UL goal documented; whether they were provided with an UL self-exercise training programme (Graded Repetitive Arm Supplementary Programme (GRASP)) " | "surveyed patients (from the audited group not receiving the device) as to the frequency with which GRASP was used and the conditions in which it was used (i.e. self-directed or supervised)" |
| Chin 2019 | “people in rehabilitation” | - | - | - | - |
| Chin2020 | - | - | - | - | - |
| de Jong 2018 | "Content of conventional therapy for the severely affected arm during subacute rehabilitation after stroke: An analysis of physiotherapy and occupational therapy practice" | “The therapists administered their interventions… in a way that was generally consistent with the recommendations of the Dutch stroke guidelines” | Appendix A  “all interventions and treatment goals were grouped under the relevant domains and chapters of the ICF through discussion until consensus was reached. The first author (LDdJ) then collated the results from the different groups …consisting of 14 ICF categories and one non-ICF category ('miscellaneous').” | Appendix A.  "Strength training of (isolated) muscle(groups) "  "Mental preparation of sequence and coordination of motor actions” | “Therapists recorded their arm interventions during or right after each treatment session” |
| Flynn 2022 | “To our knowledge this is the first study to have observed an increase in UL practice when RTUL is implemented into routine clinical practice.” | - | “UL therapy tasks were categorized as either impairment-related therapy or activity-related based on previously developed coding lists for observing UL practice by stroke survivors” | - | “All occupational therapy and physiotherapy gym-based sessions conducted in that day were observed except where the sole purpose of the session was assessment” |
| Horsley 2019 | “Participants in the control group received usual upper limb therapy only” | - | “usual upper limb therapy was recorded daily, and was categorised as either active (eg, practice of active movements); passive (eg, stretching, splinting, passive ranging, oedema management, electrical stimulation without targets or counting repetitions); functional (eg, part or whole task practice, tasks that actively included affected arm); or electrical stimulation for shoulder subluxation.” | “consisted of strengthening and task-specific practice of upper limb activities.”    “categorised as either active … or electrical stimulation for shoulder subluxation.” | “Upper limb therapy usually involved both group and individual sessions” |
| Schneider 2019 | "This paper presents the process that a rehabilitation unit went through to increase the intensity of practice undertaken within usual care upper limb rehabilitation." | “Practice was recorded against activities recommended in the stroke-guideline for upper limb activity impairment; specifically section 6.1 Amount, intensity and timing of rehabilitation, and section 6.3.5 Upper limb activity” | “such as reaching plus placing an object to the goal destination” p364    “active shoulder forward flexion in standing and returning the arm back to the side of the body.” P 364 | Overall, this would lead to more repetitions per minute of practice undertaken during cyclic electrical stimulation than the repetitions completed during task-specific    Practice was defined as the patient being actively involved in producing the upper limb movement (e.g. reach and grasp, functional electrical stimulation)    Functional electrical stimulation was included as, unlike cyclic electrical stimulation, it involves mental practice | "Occupational therapists facilitate the class for patients with upper limb activity limitations"    “In this setting, most of the upper limb intervention is provided in a group-based format in an inpatient, upper limb rehabilitation class. Occupational therapists facilitate the class" |
| Vratsistas-Curto 2021 | “All participants completed task-specific arm retraining as part of their usual rehabilitation program” | - | “motor and functional skills such as personal hygiene, grooming, feeding, and handwriting”    Supplementary  “e.g external rotation to target” | All participants completed task-specific arm retraining as part of their usual rehabilitation program during | “Independent practice on the ward, outside of therapy hours, was encouraged and set up by the treating therapist”    “Stroke survivors receive one-to-one therapy sessions as well as group-based classes to increase practice opportunities” |

**STable 4**: Results quality appraisal

| Joanna Briggs Institute - Cross sectional critical appraisal of included studies | | | | | | | | |
| --- | --- | --- | --- | --- | --- | --- | --- | --- |
| Legend  Y=Yes  N=No              Criteria | Broderick et al., 2021 | Chin et al., 2019 | Chin et al., 2020 | de Jong et al., 2018 | Flynn et al., 2022 | Horsley et al., 2019 | Schneider et al., 2019 | Vratsistas-Curto et al., 2021 |
| Were the criteria for inclusion in the sample clearly defined? | Y | Y | Y | Y | Y | Y | Y | Y |
| Were the study subjects and the setting described in detail? | Y | Y | Y | Y | Y | Y | N | Y |
| Was the exposure measured in a valid and reliable way? | Y | Y | Y | Y | Y | N | Y | Y |
| Were objective, standard criteria used for measurement of the condition? | Y | Y | Y | Y | Y | Y | N | Y |
| Were confounding factors identified? | Y | Y | Y | Y | Y | Y | N | Y |
| Were strategies to deal with confounding factors stated? | Y | Y | Y | Y | Y | Y | N | Y |
| Were the outcomes measured in a valid and reliable way? | Y | Y | Y | Y | Y | Y | N | Y |
| Was appropriate statistical analysis used? | N | Y | Y | Y | Y | Y | Y | Y |
| Total, /8 | 7/8 | 8/8 | 8/8 | 8/8 | 8/8 | 7/8 | 3/8 | 8/8 |
